# Overexpression of leptin receptor predicts an unfavorable outcome in Middle Eastern ovarian cancer

**DOI:** 10.1186/1476-4598-8-74

**Published:** 2009-09-18

**Authors:** Shahab Uddin, Rong Bu, Maqbool Ahmed, Jehad Abubaker, Fouad Al-Dayel, Prashant Bavi, Khawla S Al-Kuraya

**Affiliations:** 1 Department of Human Cancer Genomic Research, Research Center, Department of Pathology, King Faisal Specialist Hospital and Research Center, Riyadh, Saudi Arabia

## Abstract

**Background:**

Recent epidemiological studies have suggested that obesity is associated with ovarian cancer. Obesity hormone leptin and its receptor (Ob-R) contribute to tumor development by enhancing cell growth and survival. This study was design to investigate the prevalence of leptin and Ob-R in Middle Eastern epithelial ovarian cancer (EOC) and to analyze the role of leptin and the mechanisms under its action in EOC tissue sample and cell lines.

**Methods:**

The expression of leptin and Ob-R was examined by immunohistochemistry in a tissue microarray of 156 EOC samples. Proliferation of EOC cells in response to leptin was assessed by MTT assays, and its anti-apoptotic effects were determined by flow cytometry. Effect of leptin on PI3K/AKT signaling pathway was further determined by western blotting.

**Results:**

In clinical samples, Ob-R overexpression was seen in 59.2% EOCs and was significantly associated with poor progression free survival (p = 0.0032). Furthermore, Ob-R expression was associated with anti apoptotic proteins Bcl-XL (p = 0.0035) and XIAP (p = 0.0001). In vitro analysis using EOC cell lines showed that leptin stimulated cell proliferation and inhibits apoptosis via activation of PI3K/AKT signaling pathway. Inhibition of PI3K activity by LY294002, a specific inhibitor of PI3-kinase abrogated leptin mediated PI3K/AKT signaling. Gene silencing of Ob-R with Ob-R siRNA in EOC cells resulted in down regulation of phospho-AKT and its down stream targets.

**Conclusion:**

Our findings have potential clinical implication for EOC development and progression.

## Background

Despite rapid advances in understanding ovarian cancer etiology, epithelial ovarian cancer (EOC) remains the most lethal form of gynecologic cancers [1-4]. Malignant transformation of normal ovarian epithelial cells is caused by genetic alteration that disrupts proliferation, programmed cell death and senescence.

Leptin, the product of obesity gene (Ob) is suggested to be associated with cancer development and progression in many epithelial cancers including EOC [5-8]

Leptin is 16KD adipokine produced predominantly by adipocytes with wide range of biological activities including appetite regulation, bone formation, reproductive function and angiogenesis [9-11]. Leptin mediated signaling pathways play an important role in cancer cell proliferation, invasion and metastasis [5]. Leptin exerts its activity through specific membrane receptor, the obesity receptor (Ob-R), which is assigned to class I cytokine receptor family [12]. Six splice variants of Ob-R have been identified up to now; a long isoform, four short isoforms discriminated by the different lengths of intracellular domain, and the secreted isoform, which modulates blood leptin [12,13]. According to the current knowledge, leptin signaling pathway is mainly transduced by JAK/STAT, MAPK and PI3K signaling pathways [5]. Previous study [8] has suggested that leptin signaling pathway is transmitted via MAP kinase pathway. However, the interaction between leptin signaling and PI3K/AKT pathway in ovarian cancer remains unknown.

A recent epidemiological study [14] has found that among women who have never used menopausal hormone therapy, obese women are at increased risk of developing ovarian cancer compared with women of normal weight. Although a hormonal mechanism was suggested as a link between ovarian cancer and obesity, at present, a clear biological explanation for risk associated between obesity and EOC is not fully known. Therefore, the effects of obesity on ovarian cancer represent a critical intersection between these two important health problems. However, whether there is a direct relationship between leptin and ovarian cancer cannot be conclusively stated as increased leptin and ovarian cancer may both be secondary consequences of obesity. Considering the fundamental role of leptin and Ob-R in cancer development and progression, we sought to examine the expression pattern of leptin and Ob-R in large cohort of Middle Eastern EOC using TMA immunohistochemical analysis. We then examined the expression of leptin and Ob-R using EOC cell lines. Furthermore, we investigated the effect of leptin on malignant properties of EOC including proliferation and apoptosis. Finally we elucidated the PI3K/AKT signal transduction pathway regulating leptin-induced changes in the cancerous properties of EOC.

## Results

### Immunohistochemical detection of Ob-R expression and its association with clinicopathological parameters

**Figure 1:**
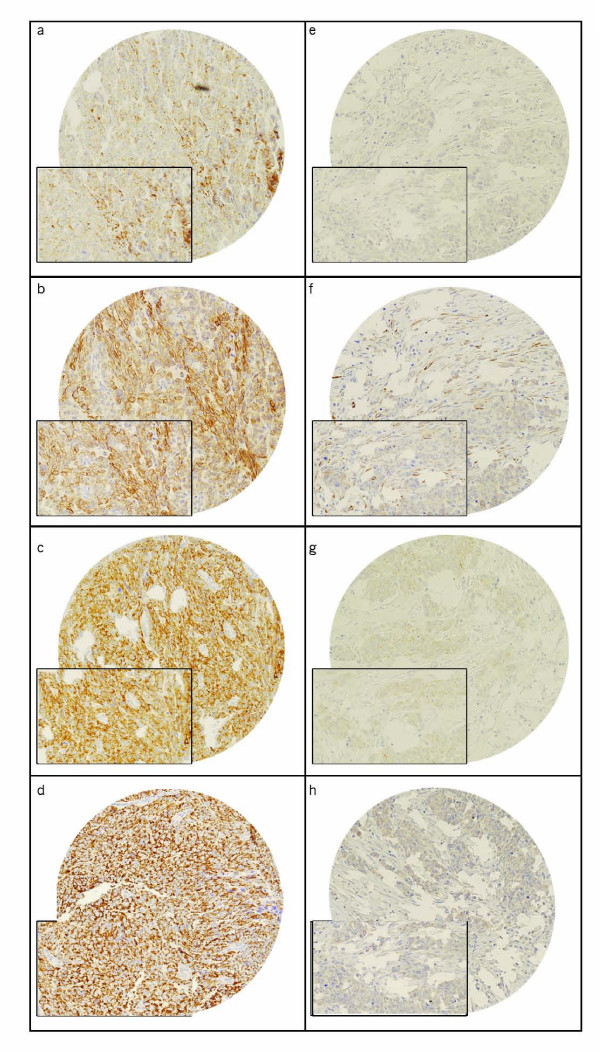
**Tissue microarray based immunohistochemical analysis of Ob-R, pGSK3, Bcl-XL and XIAP in EOC patients**. An EOC tissue microarray spot showing over expression of (a) Ob-R, (b) pGSK3, (c) Bcl-XL and (d) XIAP. In contrast, another EOC tissue microarray spot showing low expression of (e) Ob-R, (f) pGSK3, (g) Bcl-XL and (h) XIAP. 20 X/0.70 objectives on an Olympus BX 51 microscope (Olympus America Inc, Center Valley, PA, USA. With the inset showing a 40X/0.85 aperture magnified view of the same.

Ob-R expression was seen in 59.2% (90/152) of the EOCs analyzed (Figure 1). No association was observed between Ob-R overexpression and age, FIGO Stage, Histology type and grade (Table 1). Ob-R expression was linked to PI3K/AKT signaling pathway as evidenced by direct association of Ob-R expression with pGSK3 (p = 0.0009), PTEN (p = 0.0002) and end stream anti-apoptotic markers XIAP (p = 0.0001) and Bcl-XL (p = 0.0035) expression. However no association was seen with p-AKT (p = 0.2082).

**Table 1:** Correlation between Leptin-R(Ob-R) Status and clinical status in Epithelial Ovarian Carcinoma (EOC).

**Epithelial Group**			**Leptin-R high**		**Leptin-R low**		**P value**
				
	**N**	**%**	**N**	**%**	**N**	**%**	
**Total Number of Cases**	152		90	59.2	62	40.8	

**Age**							
< = 50 years	59	38.8	34	57.6	25	42.4	0.7519
>50 years	93	61.2	56	60.2	37	39.8	

**Tumour Stage**							
Stage I-II	8	5.6	5	62.5	3	37.5	0.8749
Stage III-IV	134	94.4	80	59.7	54	40.3	

**Histopathology**							
Clear cell	4	2.6	3	75.0	1	25.0	0.2909
Endometriod	21	13.8	13	61.9	8	38.1	
Serous	122	80.3	73	59.8	49	40.2	
Undifferentiated	5	3.3	1	20.0	4	80.0	

**FIGO Grade**							
Well differentiated	27	17.8	15	55.6	12	44.4	0.9031
Moderately Diff	81	53.3	49	60.5	32	39.5	
Poorly Diff	44	29.0	26	59.1	18	40.9	

**P-AKT Ser 473**							
High (2-3)	75	52.1	49	65.3	26	34.7	0.2082
Low (0-1)	69	47.9	38	55.1	31	44.9	

**pGSK3B(Ser9)**							
Above 45	116	81.7	77	66.4	39	33.6	0.0009
Below = 45	26	18.3	8	30.8	18	69.2	

**PTEN**							
0-1	33	22.9	11	33.3	22	66.7	0.0002
2-3	111	77.1	77	69.4	34	30.6	

**XIAP**							
Above 70	128	88.3	84	65.6	44	34.4	0.0001
Below = 70	17	11.7	3	17.6	14	82.4	

**Bcl-XL**							
Above 180	122	85.9	79	64.7	43	35.3	0.0035
Below = 180	20	14.1	6	30.0	14	70.0	

PFS Median (Months)^			13.1		21.0		0.0032

#of the 152 EOCs with available Ob-R expression data, FIGO stage was available only in 142 cases; in remaining stage was unknown.

$ Analysis failure of some markers for these IHC markers was attributed to missing or non representative spots

^PFS= progression free survival

### Ob-R expression and progression overall survival

**Figure 2:**
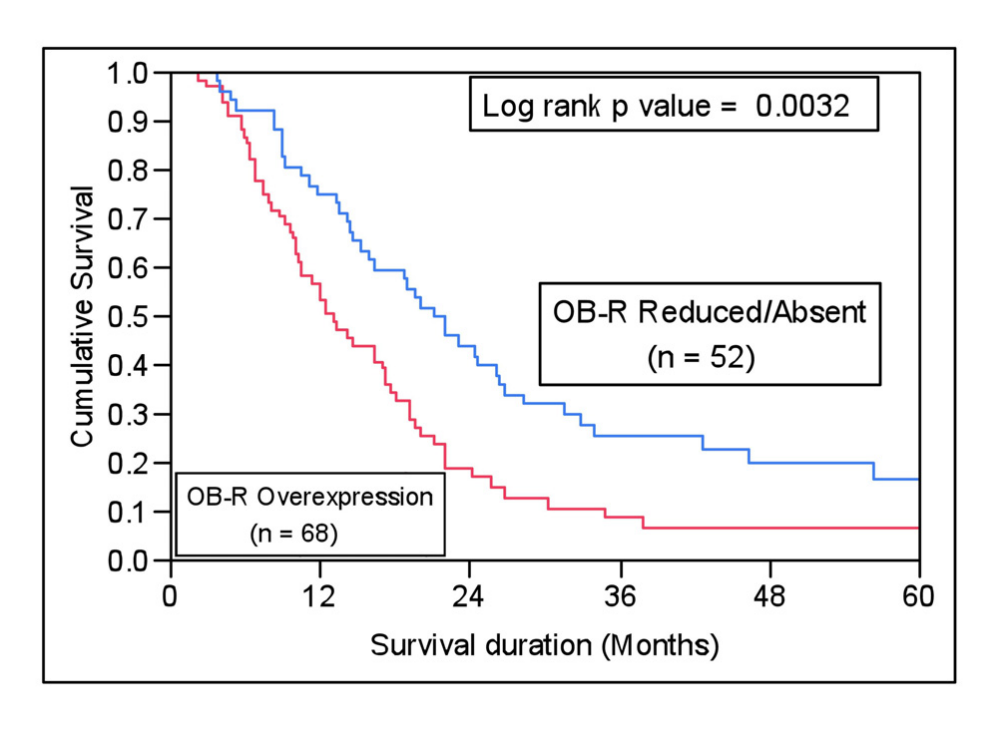
**Kaplan Meier survival analysis in EOC patients showing reduced or absent leptin-R (n = 52) and those with high expression of Ob-R (n = 68)**.

EOC patients with low expression of Ob-R had a poor progression free survival (PFS) of 13.1 months as compared to 21 months (p = 0.0032) with low Ob-R expression (Figure 2). In the multivariate analysis using Cox Proportional Hazard model for multiple factors like age, FIGO stage, grade and Ob-R expression, the relative risk was 1.96 for high Ob-R expression (95% CI 1.28-3.06; p = 0.0020) and 1.81 for AJCC stage IV (95% CI 1.08-2.93; p = 0.0243). Thus Ob-R overexpression was an independent prognostic marker for poor survival in multivariate analysis (Table 2).

**Table 2:** Univariate and Multivariate analysis of Ob-R in Epithelial Ovarian Cancer using Cox Proportional Hazard Model.

**Clinical Parameters**	**UNIVARIATE**		**MULTIVARIATE**	
	
	**Risk Ratio (95% CI)**	**P value**	**Risk Ratio (95% CI)**	**P value**
Age Above = 50	0.80 (0.54-1.19)	0.2751	0.74 (0.49 - 1.14)	0.1712
Stage IV	2.15 (1.32 - 3.41)	0.0028	1.82 (1.08 - 2.93)	0.0243
Grade Poor	1.27 (0.82-1.93)	0.5303	1.15 (0.72 - 1.79)	0.5445
Leptin R(Ob-R) Overexpression	1.82 (1.22-2.75)	0.0032	1.96 (1.28 - 3.06)	0.0020

CI: Confidence Interval

### Immunohistochemical detection of leptin expression and its association with clinicopathological parameters

Leptin expression was noted in 89.5% (128/143) of the EOCs analyzed and leptin staining was seen in the nuclear as well as cytoplasmic compartment. As shown in Table 3 leptin expression was linked to PI3K/AKT signaling pathway as evidenced by direct significant association of leptin expression with p-AKT (p = 0.0344). However no association was seen with expression of PTEN (p = 0.3096) and end stream anti-apoptotic markers XIAP (p = 0.3500) and Bcl-XL (p = 0.1724). Also leptin overexpression was not associated with patient's age, histology type, tumor grade, FIGO stage and progression free survival (Table 3).

**Table 3:** Correlation between leptin Status and clinical status in Epithelial Ovarian Carcinoma (EOC).

**Epithelial Group**			**Leptin High**		**Leptin low**		**p value**
				
	**N**	**%**	**N**	**%**	**N**	**%**	
**Total Number of Cases**	143		128	89.5	15	10.5	

**Age**							
< = 50 years	57	39.9	53	93.0	4	7.0	0.2588
>50 years	86	60.1	75	87.2	11	12.8	

**Tumour Stage**							
Stage I-II	8	6.0	7	87.5	1	12.5	0.9115
Stage III-IV	125	94.0	111	88.8	14	11.2	

**Histopathology**							
Clear cell	4	2.8	3	75.0	1	25.0	0.6630
Endometriod	21	14.7	19	90.5	2	9.5	
Serous	114	79.7	102	89.5	12	10.5	
Undifferentiated	4	2.8	4	100.0	0	0	

**FIGO Grade**							
Well differentiated	27	18.9	24	88.9	3	11.1	0.9815
Moderately Diff	75	52.5	67	89.3	8	10.7	
Poorly Diff	41	28.7	37	90.2	4	9.8	

**P-AKT Ser 473**							
High (2-3)	74	52.1	70	94.6	4	5.4	0.0344
Low (0-1)	68	47.9	57	83.8	11	16.2	

**pGSK3B(Ser9)**							
Above 45	116	81.7	103	88.8	13	11.2	0.5852
Below = 45	26	18.3	24	92.3	2	7.7	

**PTEN**							
Low (0-1)	32	22.5	27	84.4	5	15.6	0.3096
High (2-3)	110	77.5	100	90.9	10	9.1	

**XIAP**							
Above 70	124	87.9	112	90.3	12	9.7	0.3500
Below = 70	17	12.1	14	82.3	3	17.7	

**Bcl-XL**							
Above 180	122	85.9	111	91.0	11	9.0	0.1724
Below = 180	20	14.1	16	80.0	4	20.0	

PFS Median (Months)^				17.0		10.3	0.1422

# of the 143 EOCs with available Ob-R expression data, FIGO stage was available only in 133 cases; in remaining stage was unknown.

$ Analysis failure of some markers for these IHC markers was attributed to missing or non representative spots

^PFS= progression free survival

### Leptin increase proliferation of EOC cells

**Figure 3:**
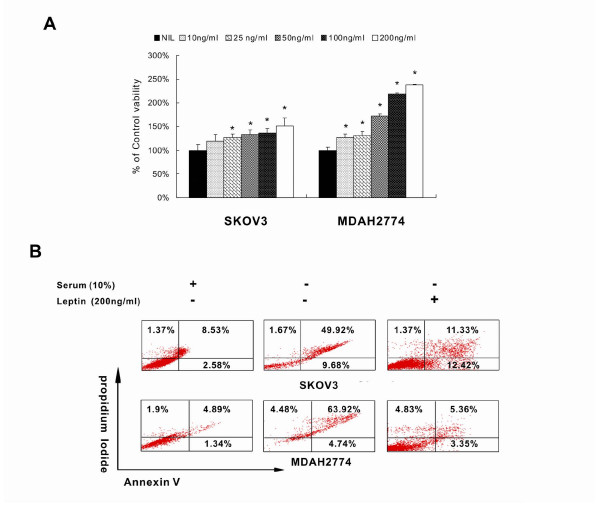
**Leptin increase proliferation of EOC cells and prevents serum starved apoptosis in EOC cells**. **(A) **SKOV3 and MDAH2774 cell lines were seeded in 96 well plates and after 24 hours serum free medium alone or with various doses of leptin as indicated was added. After 48 hours cell proliferation was measured MTT as described in materials and methods. The graph displays the mean +/-SD (standard deviation) of six independent experiments. (**B**) EOC cell lines were seeded in six well plates and after 24 hours serum free medium alone or 100 ng/ml was added. After 48 hours apoptosis was measured by annexin/PI staining.

The effects of leptin on growth rate of EOC cell lines were determined using MTT assay. MDAH2774 and SKOV3 cells were initially serum starved for 24 hours and then stimulated with various doses of recombinant leptin (10-200 ng/ml) for 48 hours compared to with cell serum free control cultures. As shown in Figure 3A, leptin induced significant cell growth of both EOC cell lines in a dose dependent manner.

### Leptin prevent serum-starved apoptosis in EOC cells

EOC cell lines were seeded in six well plates and after 24 hours serum free medium alone or 100 ng/ml leptin was added. After 48 hours, apoptosis was measured by annexin/PI staining. As shown in Figure 3B, serum starvation resulted in apoptosis. Treatment of EOC cell line with leptin significantly prevented serum starved apoptosis suggesting that leptin counteract apoptosis in EOC cells (p = 0.0005).

### Leptin activates the PI3-Kinase/AKTsignaling pathway

**Figure 4:**
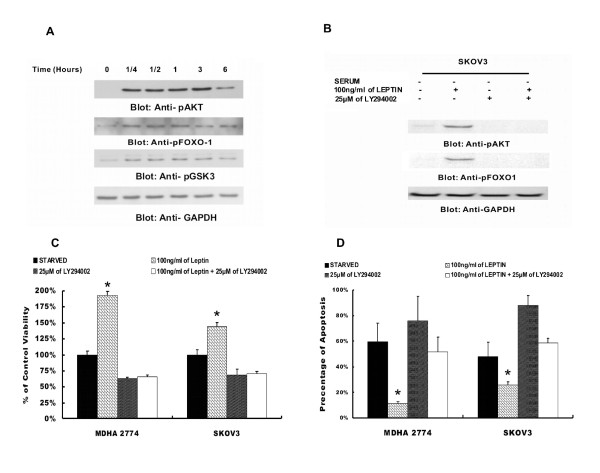
**Leptin activate PI3-kinase/AKT signaling pathway**. (**A**) MDAH2774 cells were serum starved for 24 hours in serum free medium, followed by stimulation with recombinant leptin (100 ng/ml) for various time periods as indicated. After cell lysis, 20 μg proteins were separated by SDS-PAGE, transferred to immobilon membrane, and immunoblotted with antibodies against p-AKT-Ser 473, FOXO1, and beta actin. (**B**) Inhibition of PI3-kinase/AKT pathway prevented leptin-induced activation of AKT pathway in EOC cells. SKOV3 cells were serum starved in the presence and absence of LY294002 as indicated for 48 hours and subsequently stimulated with 100 ng/ml of recombinant leptin for 3 hours, Cells were lysed and proteins were separated by SDS-PAGE, transferred to immobilon membrane, and immunoblotted with antibodies against p-AKT-Ser 473, FOXO1, and beta actin. (**C**) LY294002-inhibitor abrogate leptin-mediated cell proliferation and prevented leptin-induced anti-apoptotic effects in EOC cells. MADH2774 and SKOV3 cells were serum starved in the presence and absence of LY294002 as indicated and subsequently stimulated with 100 ng/ml of recombinant leptin for 48 hours. Cell proliferation was measured by MTT assays and (**D**) apoptosis was measured by Anenexin/PI staining. The graph displays the mean +/- SD of six independent experiments (* p < 0.05).

PI3-kinase/AKT pathways have been implicated in playing crucial roles in regulating cell growth, cell proliferation prevention of apoptosis, which altogether attribute tumorigenesis [15]. In view of these findings we sought to determine whether PI3-kinase signaling is activated during leptin stimulated EOC cell line proliferation. MDAH2774 cells were stimulated with 100 ng/ml leptin for various time periods. Cells were lysed and proteins were separated on SDS-PAGE and immunoblotted with p-AKT (activated AKT) and p-FOXO1 antibodies. As shown in Figure 4A, leptin treatment of MDAH2774 phosphorylated AKT and FOXO1 as early as 15 minutes and remained phosphorylated till 3 hours. Similar results were obtained with other EOC cell lines (data not shown). These results suggest that leptin mediated cell proliferation occurs via PI3K/AKT signaling pathway.

### Inhibition of PI3-kinase prevents leptin mediated AKT activation and its downstream effector FOXO1

Since our study suggesting that leptin stimulated PI3-kinase signaling plays a role in EOC proliferation and promotes its anti-apoptotic effects. We sought to determine whether the inhibition of PI3'-kinase by its specific inhibitor, LY294002, abrogated leptin mediated PI3K/AKT signaling in EOC cell lines. Cells were seeded on culture plates for 24 hours. Starved EOC cell were pre-treated with 20 μM LY294002 for 2 hours and subsequently treated with and without 100 ng/ml leptin for 3 hours. Cells were lysed and proteins were separated on SDS-PAGE and immunoblotted by antibodies against p-AKT and p-FOXO1. As shown in Figure 4B, leptin phosphorylated AKT and FOXO1 in MDAH2774 cell line and pre-treatment with LY294002, prevented AKT and FOXO1 phosphorylation. In addition, pre-treatment of EOC cells with LY294002, abrogated leptin-induced cell proliferation (Figure 4C) as well as prevented leptin-mediated anti-apoptotic effects (Figure 4D) on EOC cells suggesting that PI3-kinase/AKT pathway plays a critical role in leptin-induced growth and proliferation of EOC cells. These data is also suggesting that leptin is acting upstream of PI3-kinase/AKT pathway in modulating its anti-apoptotic response in EOC cells.

### EOC cell lines express leptin receptors that mediate the PI3-kinase/AKT signaling pathways

**Figure 5:**
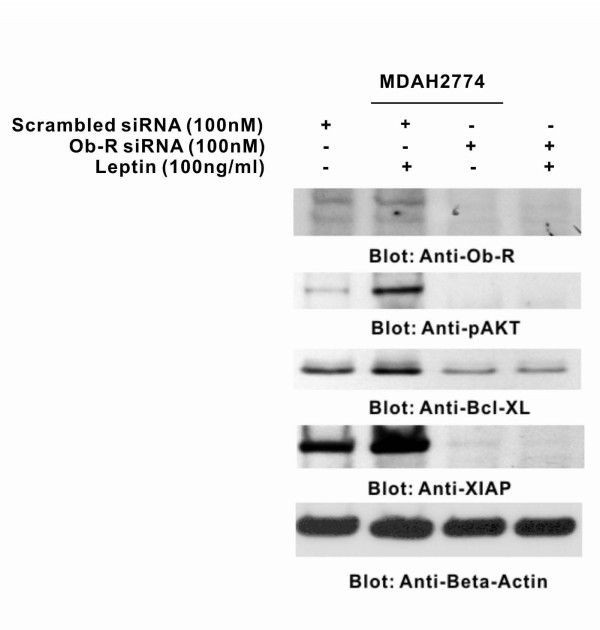
**Leptin required functional leptin receptor (Ob-R) for activation of PI3-kinase/AKT signaling in EOC cell lines**. MDAH2774 cells were transfected with scrambled siRNA and Ob-R siRNA (50 and 100 nM) with Lipofectamine. After 48 hours, cells were starved and treated with 100 ng/ml for 3 hours and proteins were immuno-blotted with antibodies against Ob-R, p-AKT-Ser473, and FOXO1, Bcl-XL, XIAP and beta actin.

To investigate whether leptin receptors are functional and linked to coordinate with PI3-kinase/AKT signaling pathway to regulate cell growth and proliferation of EOC cell lines, we utilized small interfering RNA (siRNA) strategies to transfect Ob-R specific siRNA as well as scrambled non specific siRNA in MDAH2774 cell line. After 48 hours transfection, cell were starved and then treated with and without 100 ng/ml leptin for 3 hours. As shown in Figure 5, MDAH2774 expressed functional leptin receptors, as shown previously[4] Treatment of scrambled siRNA harboring MDAH2774 cells with leptin showed activation of AKT, FOXO1 and elevated level of XIAP and Bcl-XL proteins that are involved in PI3-kinase/AKT pathway and play a critical role in cell survival. On the other hand expression of Ob-R specific siRNA in MDAH2774 cells knocked down the expression of Ob-R and prevented leptin-induced activation of AKT, FOXO1 as well as abrogated the expression of XIAP and Bcl-XL proteins. These data suggests that leptin utilizes PI3-kinase/AKT signal transduction pathway in mediation of EOC cell proliferation, further confirming that leptin is acting upstream of PI3K/AKT pathway.

## Discussion

Recent reports suggest that leptin is overexpressed in various cancer cells and plays a role in the development and/or progression of variety of variety of malignancies including colon, gastric, endometrial and breast cancers [16-20]. These findings were further supported by experimental evidence that leptin can stimulate growth and prevent apoptosis in different cellular cancer models [21-23]. A very recent epidemiological study has shown that obesity is a risk factor for ovarian cancer in postmenopausal women [14]. High levels of serum estrogen, derived from increased adipose tissue are considered to contribute to the pathogenesis of ovarian cancer in those obese patients. However, circulating leptin is an essential factor regulating fat metabolism, it can be hypothesized that leptin itself might be involved in the development of ovarian cancer.

Therefore, we first examined the prevalence of leptin and its receptor Ob-R expression in 156 Saudi EOC samples. Our data shows that Ob-R protein was detected in 59.2% examined EOCs. Interestingly, patients with high Ob-R expression tumors showed a significant poor disease free survival (p = 0.0032) compared with reduced Ob-R expression which support the proposed role of leptin signaling in the EOC. Furthermore, Ob-R overexpression was an independent prognostic variable to predict poor progression free survival. Interaction between leptin/Ob-R and other signaling pathways such as PI3K/AKT and MAP kinase have been reported in oncogenesis of various tumors [5,8,22,24-26]. Therefore, we examined the relationship between the expression of Ob-R and the PI3K/AKT pathway protein targets in EOC. Activated AKT protein expression was seen in 52% of our EOC examined. However, no correlation was observed with Ob-R expression and AKT activation by IHC staining which might suggest the presence of other upstream signaling pathways can also be involved in the activation of AKT. However Ob-R overexpression was correlated with pGSK3 expression (p = 0.0009), PTEN (p = 0.0002) and other down-stream targets of PI3K/AKT, the anti apoptotic markers Bcl-XL and XIAP (p = 0.0035 and p = 0.0001 respectively). In addition leptin expression was seen in 89.5 of the EOCs was linked to PI3K/AKT signaling pathway as evidenced by direct significant association of leptin expression with p-AKT. However leptin expression was not associated with any clinicopathological parameters including progression free survival. BMI data was available in 100 patients and no association was noted between leptin expression by IHC and BMI. (p = 0.1260; data not shown). Thus we can hypothesize that intratumoral intracellular leptin unlike serum leptin levels are not associated with body mass index

Furthermore, in vitro analysis using EOC cell lines was conducted to study the affect of leptin on EOC cell growth. Our experiments clearly showed that leptin has oncogenic affect on EOC cells and this oncogenic effect is due to a combination of cell proliferation and inhibition of apoptosis by leptin. To elucidate the signaling pathways involved in leptin mediated induction of cancerous properties of EOC cells, we examined the effect of leptin in activation of the PI3K/AKT pathway. Our data showed that leptin rapidly stimulates the PI3K/AKT pathway and induced phosphorylation of AKT thus activating this key signal transduction pathway associated with cell growth. In addition, prevention of leptin-induced activation of PI3K/AKT with pharmacological inhibition in turn significantly reduced the activation of AKT. In addition our data showed that gene silencing of Ob-R in EOC cell lines abrogated the AKT activation as well as the expression of anti-apoptotic genes, XIAP and Bcl-XL suggesting that leptin mediated EOC growth is due to modulation of growth and anti-apoptotic genes.

## Conclusion

Our data suggests that leptin pathway might play a major role in Middle Eastern EOC, and deciphered the molecular mechanisms responsible for leptin mediated EOC cell proliferation, establishing direct association between obesity and EOC carcinogenesis and presenting involvement of key molecules of oncogenic PI3K signaling pathway.

## Methods

### Patient selection

156 patients with ovarian carcinoma diagnosed between 1991 to 2007 were selected from the files of the King Faisal Specialist Hospital and Research Centre. All samples were analyzed in a tissue microarray (TMA) format. The Institutional Review Board of the King Faisal Specialist Hospital & Research Centre approved the study.

The patients included in this study had their diagnosis, treatment and follow-up care in the departments of Obstetrics and Gynecology and Oncology at King Faisal Specialist Hospital and Research Centre. The histological subtype of each ovarian tumor sample was determined according to established criteria [27], the distribution of tumors by histological type was as follows: 125 serous (80.1%), 22 endometrioid (14.1%), 4 clear cell (2.6%) and 5 undifferentiated/mixed Epithelial (3.2%). The median age of the patient population was 56 years with a range from 19-86 years. The majority of patients underwent primary surgical staging or cytoreduction. In some patients who were not fit for primary surgery, primary neoadjuvant chemotherapy was followed by interval debulking surgery. The distribution by FIGO stage at diagnosis was: stage I-II in 8 patients (5.1%), stage III-IV in 137 (87.8%), and unknown in 11(7.1%). The median follow-up time was 14.9 months (range, 1-130 months). Progression free survival was computed from date of surgery for patients who underwent primary cytoreduction and from date of diagnosis by biopsy or cytology in those who underwent primary neoadjuvant chemotherapy. Since the majority of patients are lost to follow-up as their disease reaches its terminal stages, it was impossible to determine overall survival in this specific patient population.

### Tissue Microarray (TMA) Construction

Tissue microarrays were constructed from formalin-fixed, paraffin-embedded ovarian cancer specimens as described previously [28]. Tumor regions were mapped by a pathologist for coring. The tissue microarray was constructed with 0.6-mm diameter cores spaced 0.8 mm apart using a modified Tissue Microarrayer (Beecher Instruments, Sun Prairie, WI, USA). The tissue microarray block was cut into 5 mm sections, adhered to the slide by an adhesive tape-transfer method (Instrumedics Inc., Hackensack, NJ, USA) and UV cross linked.

### Antibodies and immunohistochemistry

Immunohistochemical studies on formalin-fixed, paraffin-embedded tissue sections were performed as describer in earlier studies [28,29]. Primary antibodies used, their dilutions, and other information is listed in Table 4. For antigen retrieval, Dako Target Retrieval Solution pH 6.0 (Catalogue number S1700) was used, and the slides were microwaved at 750W for 5 minutes and then at 250W for 30 minutes. The sections were incubated overnight with Ob-R) and the Dako Envision Plus System kit was used as the secondary detection system with DAB as chromogen. We used a mouse monoclonal antibody from Santa Cruz Biotechnology; Clone B3 to detect Ob-R expression and this antibody binds to both short and long forms of Ob-R. Similarly, leptin expression was detected by using a rabbit monoclonal antibody Y20 from from Santa Cruz Biotechnology. IHC for p-AKT was performed by staining 3-4 micrometer thick tissue micro array sections with the p-AKT (Ser 473) antibody [Survival Marker: Signal Stain Phospho-AKT (Ser 473) IHC detection kit Product No 8100 Cell Signaling Technology, Beverly, MA]. The IHC protocol included with the kit was followed with no modifications. Incubating the tissue in blocking solution blocked nonspecific binding. Endogenous peroxidase activity was quenched using peroxidase quench supplied along with the kit. Endogenous biotin was blocked and all slides were counterstained with hematoxylin, dehydrated, cleared, and cover slipped with premount. Only fresh cut TMA slides were stained simultaneously to minimize the influence of slide aging and maximize repeatability and reproducibility of the experiment. Two types of negative controls were used. One was the negative control in the kit in which the primary antibody was omitted. A preabsorption experiment using p-AKT Ser 473 blocking peptide (Cell Signaling Technology, Beverly, MA, Product No 1140) was used as the second negative control

**Table 4:** Antibodies used for tissue micro array Immunohistochemical analysis.

**Antibody**	**Clone**	**Company**	**Source**	**Dilution** **O/N@**	**Retrieval**	**Sub cellular ** **Localization**	**Detection System**
Leptin-R	B-3	SCBT	Mouse monoclonal	1:20 O/N	pH9, Pressure cooker	Cytoplasmic	EnVision+
Leptin	Y20	SCBT	Rabbit monoclonal	1:1000 O/N	pH6, Pressure cooker	Nuclear & Cytoplasmic	EnVision+
PTEN	6H2-1	Cascade Bioscience	Mouse monoclonal	1:100	pH9, Pressure cooker	Cytoplasmic	EnVision+
pAKT	Ser473	Cell Signalling	Rabbit polyclonal	Predilute O/N	pH9, Microwave	Nuclear & Cytoplasmic	Survival Marker; signal stain IHC detection kit
XIAP	48	BD Transduction	Mouse monoclonal	1:300 O/N	pH9, Microwave	Cytoplasmic	EnVision+
BCLXL	54H6	Cell Signalling	Rabbit polyclonal	1:800 O/N	pH9, Microwave	Cytoplasmic	EnVision+
pGSK3	Ser21/9	Cell Signalling	Rabbit polyclonal	1:50 O/N	pH9, Pressure cooker	Cytoplasmic	EnVision+

@overnight incubation of primary antibody

### Immunohistochemistry Assessment

In this study, Ob-R expression was categorized by doing an H score, which combines intensity of staining in each cell and percentage of stained cells. In brief, each TMA spot was assigned a staining intensity score from 0-3 [I0, I1-3], and a percent of stained tumor cells that was recorded in 5% increments from a range of 0-100 (P0, P1-3). For each spot analyzed, a score was generated from the product of intensity and percent of tumor cells stained. A final H score (range 0-300) was obtained by adding the sum of individual scores obtained for each tissue microarray spot. (H score = I1XP1+I2XP2+I3XP3). Ovarian tumors were categorized into 2 groups based on H score and using the X-tile plat as described below.

X-tile plots are constructed for assessment of biomarker and optimization of cut off points based on outcome [30,31]. The X-Tile plots allow determination of an optimal cut point while correcting for the use of minimum P statistics. Using the X-Tile program, an optimal cut point for Ob-R expression was determined at 20, with a Miller-Seigmund p value of 0.5950 as determined by X-Tile. Tumors with H score <20 were classified as low expressers (n = 62; 40.8%), and those with H score >20 were classified as high expressers (n = 90; 59.2%; Figure 1a and 1e). Similarly X-Tile was used to determine a cut point for leptin, Bcl-XL, XIAP, PTEN and pGSK3.

p-AKT & PTEN scoring was done as described earlier [32,33]. Briefly, p-AKT was scored as levels on an intensity scale ranging from 0 to 3. Scoring was performed as follows: 0, no appreciable staining in tumor cells; 1, barely detectable staining in tumor cells; 2, appreciable staining of moderate intensity, distinctly marking tumor cells and 3, readily appreciable staining of strong intensity. For purposes of statistical analysis, all cases staining at level 0 or 1 were grouped as p-AKT negative and all cases staining at level 2 and level 3 were grouped as p-AKT positive.

### Statistical Analysis

The JMP7 (SAS Institute, Inc., Cary, NC) software package was used for data analyses. Survival curves were generated using the Kaplan-Meier method, with significance evaluated using the Mantel-Cox log-rank test. Risk ratio was calculated using the Cox Proportional Hazard model in both univariate and multivariate analyses. Chi-square tests were used to examine relationship between nominal variables. The limit of significance for all analyses was defined as a p-value of 0.05.

### Cell culture

Ovarian cancer cell line, were used: MDAH2774 and SKOV3 cells were cultured in RPMI 1640 medium supplemented with 10% (vol/vol) fetal bovine serum (FBS), 100 U/ml Penicillin and 100 U/ml Streptomycin at 37°C in humidified atmosphere containing 5% CO_2_. All experiments were performed in RPMI 1640 containing 5% serum.

### Reagents and antibodies

Leptin and 3-(4, 5-Dimethylthiazol-2-yl)-2, 5-Diphenyltetrazolium Bromide (MTT) assays were purchased from Sigma (St. Louis MO, MA). Ob-R antibody was purchased from Abcam (Cambridge, United Kingdom). Antibodies against phospho-AKT, phospho-FKHR/FoxO1 antibodies were purchased from Cell Signaling Technologies (Beverly, MA, USA). Beta-actin antibody was purchased from Santa Cruz Biotechnology, Inc. (Santa Cruz, CA, USA). Annexin V kit was purchased from Molecular Probes (Eugene OR, USA). Apoptotic DNA-ladder kit was obtained from Roche (Penzberg, Germany).

### 3-(4, 5-Dimethylthiazol-2-yl)-2, 5-Diphenyltetrazolium Bromide Assays

10^4 ^cells were incubated in triplicate in a 96-well plate in the presence or absence of indicated test doses of leptin in a final volume of 0.20 ml for 48 hour. The ability of leptin to induce cell growth was determined by MTT cell proliferation assays, as previously described [34]. Replicates of 6 wells for each dosage including vehicle control were analyzed for each experiment.

### Annexin V/Propidium Iodide Dual Staining

EOC cell lines were treated with the indicated concentrations of leptin in conditions treated as indicated in Figure legends. The cells were harvested and the percentage of cells undergoing apoptosis was measured by flow cytometry after staining with fluorescein-conjugated AnnexinV/propidium iodide as previously described [35].

### Cell lysis and Immunoblotting

Cells were treated either with leptin or leptin in combination with PI3-kinase inhibitor LY294002 described in the legends and lysed as previously described [36]. Proteins (15-20 μg) were separated by SDS-PAGE and transferred to polyvinylidene difluoride (PVDF) membrane (Immobilon, Millipore, Billerica, MA). Immunoblotting was done with different antibodies and visualized by the enhanced chemiluminescence (Amersham, Piscataway, NJ) method.

### Gene silencing using small interfering RNA (siRNA)

Leptin receptor siRNA and scrambled control siRNA were purchased from Qiagen (Valencia, CA, USA). Cells were transfected using Lipofectamine 2000 (Invitrogen, Carlsbad, CA) and siRNA as described earlier [37]. After transfection for 6 hours, the lipid and siRNA complex was removed and fresh growth medium was added and incubated for 48 hours. Cells were then treated with leptin as indicated and after lysis protein levels were determined by Western Blot analysis with specific antibodies.

## Competing interests

The authors declare that they have no competing interests.

## Authors' contributions

SU designed research, performed experiments, analyzed data, and wrote the paper, RB performed experiments and analyzed data, MA performed experiments, JA performed experiments, FA provided clinical samples and data for performance of experiments and validation of data, PB performed experiments, analyzed data and helped in writing the paper, KSA designed research, analyzed data, and wrote the paper.
